# Right Ventricular Remodeling and Dysfunction in Obstructive Sleep Apnea: A Systematic Review of the Literature and Meta-Analysis

**DOI:** 10.1155/2017/1587865

**Published:** 2017-07-26

**Authors:** Abdirashit Maripov, Argen Mamazhakypov, Meerim Sartmyrzaeva, Almaz Akunov, Kubatbek Muratali uulu, Melis Duishobaev, Meerim Cholponbaeva, Akylbek Sydykov, Akpay Sarybaev

**Affiliations:** ^1^Department of Mountain and Sleep Medicine and Pulmonary Hypertension, National Center of Cardiology and Internal Medicine, Bishkek, Kyrgyzstan; ^2^Kyrgyz-Indian Mountain Biomedical Research Center, Bishkek, Kyrgyzstan; ^3^Department of Internal Medicine, University of Giessen and Marburg Lung Center (UGMLC), Justus Liebig University of Giessen, Giessen, Germany

## Abstract

**Background:**

Recent studies have reported that obstructive sleep apnea (OSA) patients present alterations in right ventricular (RV) structure and function. However, large randomized controlled trials evaluating the impact of OSA on the right ventricle are lacking.

**Methods:**

A comprehensive electronic database (PubMed, Web of Science, and Google Scholar) and reference search up to October 30, 2016, was performed. A systematic review and meta-analysis were performed to assess RV structure and function in OSA patients based on conventional echocardiography and tissue Doppler imaging.

**Results:**

Twenty-five studies with 1,503 OSA patients and 796 controls were included in this study. OSA patients exhibited an increase in RV internal diameter (weighted mean difference (WMD) (95% confidence intervals (CIs)) 2.49 (1.62 to 3.37); *p* = 0.000) and RV wall thickness (WMD (95% CIs) 0.82 (0.51 to 1.13); *p* = 0.000). Furthermore, OSA patients had a significantly elevated RV myocardial performance index (WMD (95% CI) 0.08 (0.06 to 0.10); *p* = 0.000), decreased RV S' (WMD (95% CI) −0.95 (−1.59 to −0.32); *p* = 0.003), tricuspid annular plane systolic excursion (WMD (95% CI) −1.76 (−2.73 to −0.78); *p* = 0.000), and RV fractional area change (WMD (95% CI) −3.16 (−5.60 to −0.73); *p* = 0.011).

**Conclusion:**

OSA patients display RV dilatation, increased wall thickening, and altered RV function.

## 1. Introduction

Obstructive sleep apnea (OSA) is characterized by repetitive episodes of complete and/or partial interruption of the respiratory airflow during sleep, leading to oxygen desaturation and chronic intermittent hypoxia. OSA is highly prevalent in the general population, affecting at least 9–15% of middle-aged adults [1, 2]. OSA represents a growing healthcare problem, as it has been increasingly implicated in the causation or promotion of various cardiovascular diseases [3]. Earlier reports have demonstrated increases in pulmonary artery pressure (PAP) during sleep, which suggested the development of sustained pulmonary hypertension in patients with OSA [4, 5]. Indeed, studies have shown that daytime pulmonary hypertension occurs in 20% to 40% of patients with OSA and concomitant pulmonary or heart disease [6–8]. Moreover, permanent pulmonary hypertension may also develop in OSA patients in the absence of other known cardiopulmonary disorders [9, 10]. Although pulmonary hypertension in OSA is usually mild to moderate, it confers functional limitations and a poor prognosis [11]. Furthermore, pulmonary hypertension in OSA patients can lead to the development of right ventricular (RV) hypertrophy and dysfunction. However, radionuclide ventriculography studies have demonstrated that RV dysfunction may develop independently of pulmonary hypertension in these patients [12]. Importantly, alterations in RV structure and function have been shown to predict the clinical outcomes in cardiopulmonary diseases [13, 14].

Echocardiography is a noninvasive, low-cost, time-saving, and accurate tool for assessments of alterations in cardiac structure and function. Therefore, in clinical practice, ventricular structure and function are commonly assessed using echocardiography. In recent years, several echocardiographic studies have reported that OSA patients frequently present structural and functional alterations of the right ventricle [16–21, 23, 24, 26, 29, 31–36, 38]. However, since most of the studies were small and assessed few echocardiographic parameters, the outcomes from these studies have been inconsistent. Because large randomized controlled trials directly evaluating the impact of OSA on the structural and functional alterations of the right ventricle are lacking, we aimed to perform a systematic review of the literature and meta-analysis of studies based on the conventional echocardiographic assessment of RV structure and function in OSA patients.

## 2. Methods

### 2.1. Search Strategy and Literature Screening

**Figure 1:**
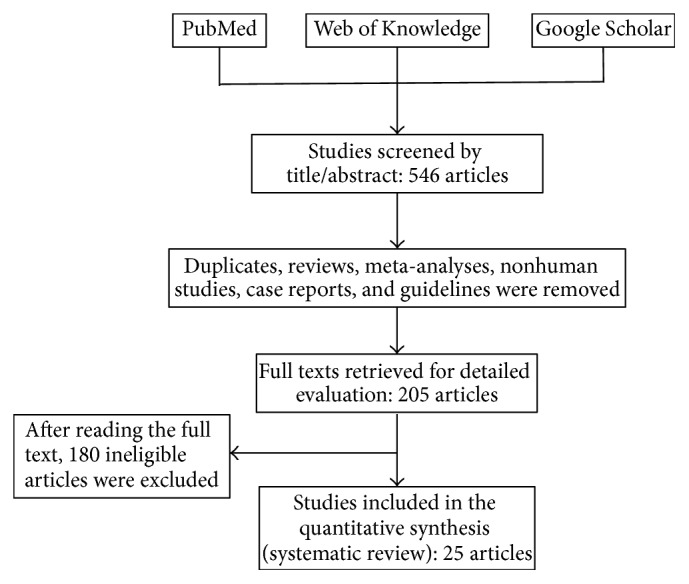
Flow chart of the literature search and study selection.

A systematic literature review was conducted using electronic databases (PubMed, Web of Science, and Google Scholar). All databases were searched up to October 30, 2016, using the following terms: “obstructive sleep apnea”, “sleep-disordered breathing”, “right ventricular function”, “right ventricular dysfunction”, “right heart failure”, and “echocardiography”. In addition, we reviewed the reference lists from all relevant articles to find other potential sources. The process of selecting studies is outlined in Figure 1.

Articles were first screened by title and abstract, and reviews, guidelines, letters, case reports, editorials, and in vitro and animal studies were excluded. In a second screen, the following criteria were used to identify potentially suitable studies: (1) studies were written fully in English; (2) enrolled subjects were adults, above 18 years old; (3) OSA patients did not have major comorbidities; (4) the study included a control group; (5) OSA was diagnosed by polysomnography; (6) the study reported at least one of the measures of RV function and RV remodeling. After the abovementioned screening, the authors obtained the full-text articles and read them carefully and independently.

### 2.2. Data Extraction and Processing

Two independent reviewers (Abdirashit Maripov and Argen Mamazhakypov) screened and extracted data from full-text articles using standardized data extraction sheets. The following study characteristics were extracted from the articles: first author, publication year, country, numbers of patients and healthy controls, mean age, sample size, body mass index (BMI), mean apnea-hypopnea index (AHI), PAP values, and RV morphology and function parameters. The main echocardiographic parameters involved in the assessment of RV morphology and function were RV internal diameter (RVID), RV wall thickness (RVWT), RV fractional area change (FAC), RV myocardial performance index (MPI), pulsed-wave tissue Doppler imaging- (TDI-) derived velocity of the tricuspid annular systolic motion (RV S'), and tricuspid annular plane systolic excursion (TAPSE). In cases of conflicting evaluations, disagreements were resolved through discussion between the authors.

### 2.3. Data Analysis

Several studies stratified patients based on OSA severity (mild, moderate, or severe) and reported grouped RV function data within each stratum. In those cases, because they represented independent samples, we considered each stratum as a separate substudy. All analyses were calculated using the statistical package Stata, version 14.1 (StataCorp, College Station, TX, USA) with the random-effects model. The weighted mean difference (WMD) with 95% confidence intervals (CIs) was calculated using a random-effects model. Fisher's *z* test was used to determine the statistical significance of the pooled WMDs. Cochran's *χ*^2^ test and the *I*^2^ statistic were used to assess between-study heterogeneity. Heterogeneity was considered statistically significant at *p* < 0.10 and *I*^2^ > 50%.

## 3. Results

### 3.1. Literature Search

Using electronic searches of the databases, 546 citations were obtained. We screened the studies through title and abstract review and removed duplicates, reviews, meta-analyses, nonhuman studies, case reports, and guidelines. Two hundred and five studies underwent full-text review. After full-text review, 180 studies were excluded according to the inclusion and exclusion criteria described above. A total of 25 studies with 1,503 OSA patients and 796 healthy control participants were included in this study. The flow diagram of the selection process is shown in Figure 1.

### 3.2. Characteristics of the Included Studies

The publication years of the involved studies ranged from 1992 to 2016. The majority of the studies (12 studies, 48%) were conducted in Turkey; 4 studies were conducted in North America (USA and Canada), 3 studies were conducted in China, and 6 studies were conducted in Europe (Belgium, Germany, Poland, and Italy). In all studies, OSA was diagnosed by polysomnography. In the control groups, OSA was excluded based on polysomnography in 18 studies. In 7 studies, healthy control subjects were enrolled based on the absence of clinical symptoms of sleepiness [17, 19, 20, 25, 29, 31] or cardiovascular diseases [39]. For the assessment of RV remodeling, 16 studies (64%) used RVID and 9 studies (36%) used RVWT. For the assessment of RV function, 11 studies (44%) used TAPSE, 14 studies (56%) used RV MPI, 6 studies (24%) used RV FAC, and 14 studies (48%) used RV S'. The main characteristics of the studies in this systematic review are listed in Table 1.

### 3.3. Meta-Analysis

**Figure 2:**
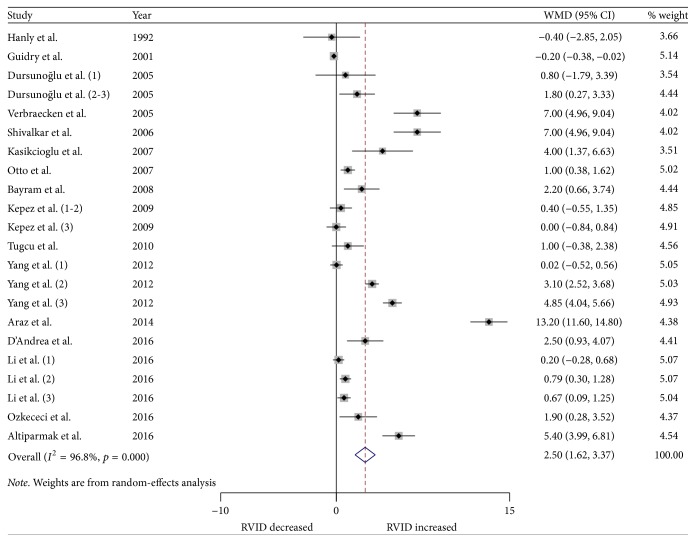
Forest plot of the differences in right ventricular internal diameter (RVID) at diastole between the OSA patients and the healthy controls based on echocardiography. OSA: obstructive sleep apnea; WMD: weighted mean difference; CI: confidence interval.

**Figure 3:**
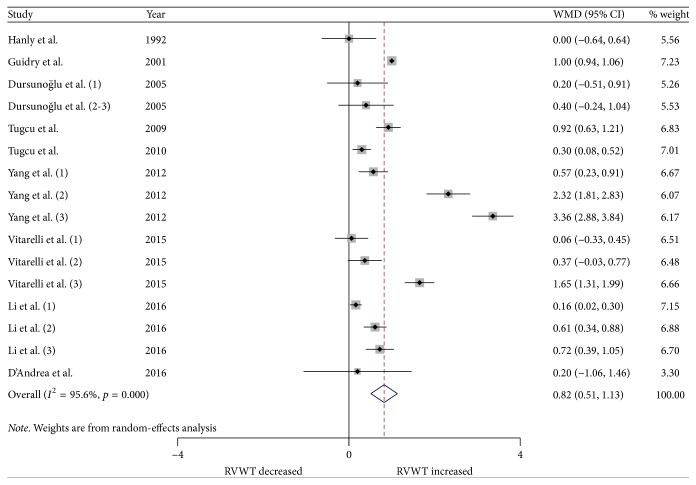
Forest plot of the differences in right ventricular free wall thickness (RVWT) between the OSA patients and the healthy controls based on echocardiography. OSA: obstructive sleep apnea; WMD: weighted mean difference; CI: confidence interval.

**Figure 4:**
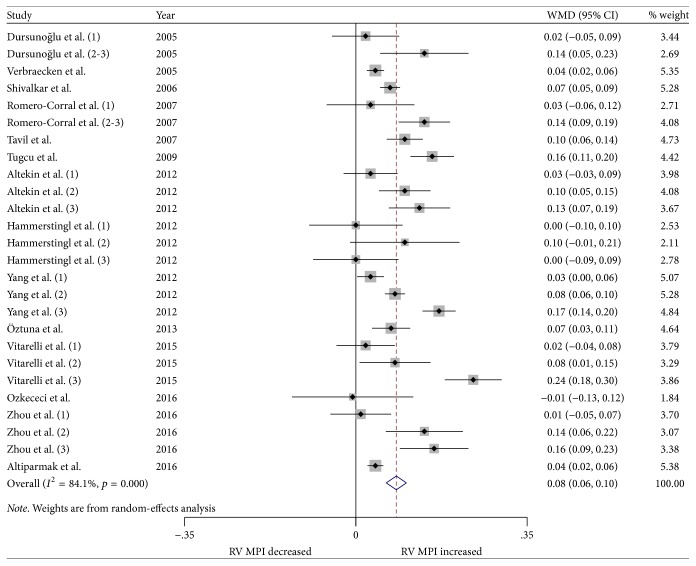
Forest plot of the differences in right ventricular myocardial performance index (RV MPI) between the OSA patients and the healthy controls based on echocardiography. OSA: obstructive sleep apnea; WMD: weighted mean difference; CI: confidence interval.

**Figure 5:**
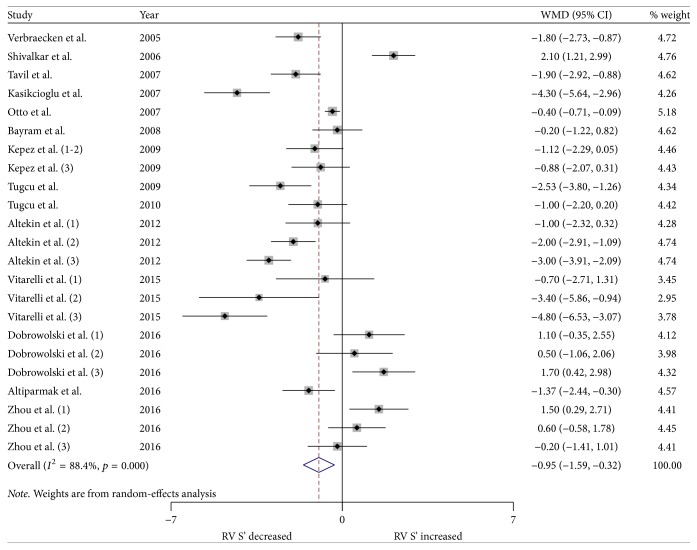
Forest plot of the differences in right ventricular annular systolic velocity (RV S') between the OSA patients and the healthy controls based on echocardiography. OSA: obstructive sleep apnea; WMD: weighted mean difference; CI: confidence interval.

**Figure 6:**
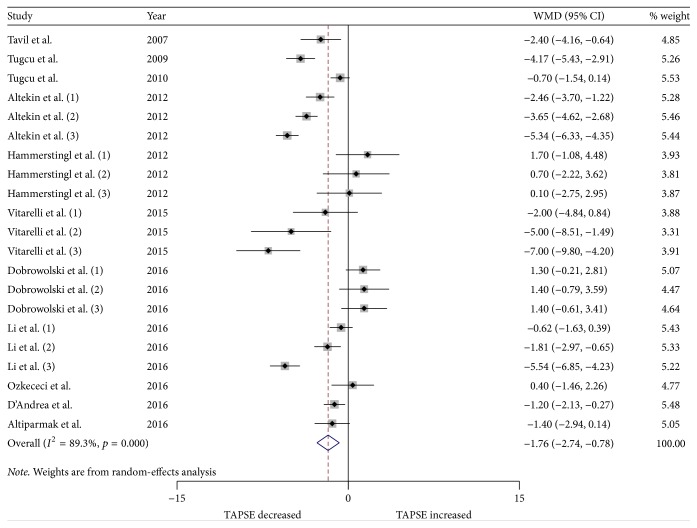
Forest plot of the differences in tricuspid annular plane systolic excursion (TAPSE) between the OSA patients and the healthy controls based on echocardiography. OSA: obstructive sleep apnea; WMD: weighted mean difference; CI: confidence interval.

**Figure 7:**
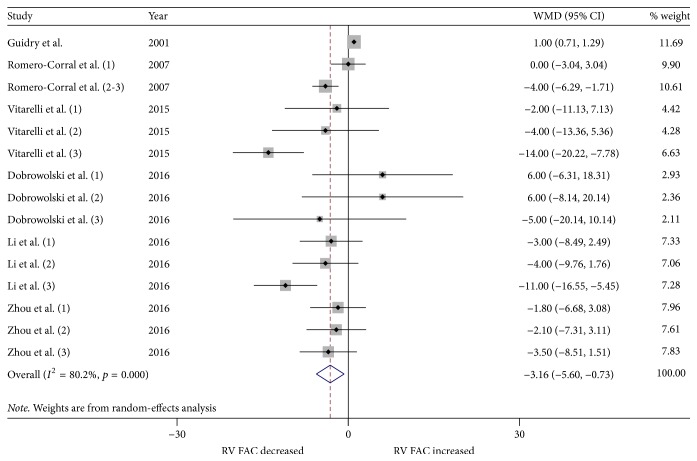
Forest plot of the differences in right ventricular fractional change (RV FAC) between the OSA patients and the healthy controls based on echocardiography. OSA: obstructive sleep apnea; WMD: weighted mean difference; CI: confidence interval.

We performed a meta-analysis for changes in RV structure and function. Figures 2 and 3 show the changes in RVWT and RVID. Changes in RV MPI, RV S', TAPSE, and RV FAC are shown in Figures 4–7.  Table 2 shows the summary data of all parameters of the RV structure and function determined by the meta-analysis. Heterogeneity was obvious in the assessment of the RV structure and function parameters, which could have been a result of differences in geographical location, participants' ages, severities of disease, and RV remodeling.

#### 3.3.1. RV Remodeling

To evaluate RV structure, RVID and RVWT were assessed. Changes in RVID were reported in 16 studies (22 strata) involving 902 OSA patients and 596 control subjects. The meta-analysis showed that the RVID in patients with OSA was significantly larger compared to the controls (WMD (95% CIs) 2.49 (1.62 to 3.37); *p* = 0.000; Figure 2). In addition, changes in RVWT were reported in 9 studies (16 strata) involving 579 patients with OSA and 379 control subjects. Patients with OSA had significantly increased RVWT compared to the controls (WMD (95% CIs) 0.82 (0.51 to 1.13); *p* = 0.000; Figure 3).

#### 3.3.2. RV Function

To evaluate RV function, RV MPI, RV S', TAPSE, and RV FAC were assessed. The most commonly reported RV functional parameters were RV MPI (14 studies, 26 strata, 864 patients with OSA and 434 control subjects) and RV S' (14 studies, 23 strata, 639 patients with OSA and 391 control subjects). The TAPSE was reported in 11 studies (21 strata, 655 patients with OSA and 378 control subjects), while 6 studies assessed the RV FAC (15 strata, 422 patients with OSA and 239 control subjects). The meta-analysis demonstrated that the RV MPI was significantly elevated in patients with OSA compared to the controls (WMD (95% CI) 0.08 (0.06 to 0.10); *p* = 0.000; Figure 4). We also found significant differences between the patients with OSA and controls for RV S' (WMD (95% CI) −0.95 (−1.59 to −0.32); *p* = 0.003; Figure 5). In addition, TAPSE (WMD (95% CI) −1.76 (−2.73 to −0.78); *p* = 0.000; Figure 6) and RV FAC (WMD (95% CI) −3.16 (−5.60 to −0.73); *p* = 0.011; Figure 7) were also significantly decreased in the patients with OSA compared to the controls.

## 4. Discussion

To our knowledge, this is the first meta-analysis to systematically evaluate changes in RV structure and function in OSA patients. In this meta-analysis, 25 case-control studies were included, with a total of 1,503 patients with OSA and 796 healthy controls. In the present study, we found that alterations in echocardiographic parameters of RV remodeling, including an increase in RVWT and RVID, were important features in OSA patients without major comorbidities. Furthermore, our meta-analysis demonstrated that, in these patients, conventional echocardiography and pulsed-wave TDI-derived parameters of RV function were significantly altered compared to the controls. We found significant heterogeneity between studies. However, heterogeneity is to be expected given the variety of studies conducted by different teams at various geographic locations, the variation in disease severity, and differences in the patient populations. In most studies, the investigators were blinded to the participants' study group status [15, 16, 21–27, 29, 31, 33–35, 38, 39]. In addition, interobserver agreement and intraobserver reproducibility were assessed in most of the studies [15, 16, 21, 23–25, 27, 29, 33, 36, 38, 39].

Although cardiac magnetic resonance imaging is considered as the gold standard for morphological and functional assessment of the right ventricle, conventional echocardiography remains the first-resort imaging modality in routine clinical practice due to its low cost, simplicity, reproducibility noninvasive nature, safety, and lack of ionizing radiation [40, 41]. However, accurate evaluation of RV morphology and function remains challenging in clinical practice due to its complex geometric shape [42]. Nevertheless, echocardiography was performed according to the recommendations of the American Society of Echocardiography in all of the studies included in the current meta-analysis [42].

Conventional echocardiography and pulsed-wave TDI allow for the assessment of RV function by quantifying RV FAC, MPI, TAPSE, and RV S'. Several studies exploiting conventional echocardiography and tissue Doppler imaging have reported that OSA patients frequently present structural and functional alterations of the right ventricle [16–21, 23, 24, 26, 29, 31–36, 38]. In contrast, other studies did not reveal any changes in RV morphology and function in OSA patients by the application of conventional echocardiography and TDI [15, 22, 25, 27, 30, 37, 39]. Importantly, novel techniques, including 2D and 3D speckle-tracking echocardiography, are very sensitive and can reveal alterations in RV morphology and performance in early disease stages when changes in the conventional echocardiographic parameters are not yet present [25, 27, 36, 39]. However, these sophisticated techniques require expertise and have not been extensively validated for the assessment of RV function.

Multiple mechanisms underlie alterations in RV morphology and function in OSA patients [43]. Numerous studies have reported permanent pulmonary hypertension in OSA patients. In this population, pulmonary hypertension is often associated with chronic obstructive pulmonary disease [6, 7]. In addition, OSA patients with pulmonary hypertension often have cardiac comorbidities, including hypertension and left ventricular dysfunction. However, permanent pulmonary hypertension may also develop in OSA patients in the absence of other known cardiopulmonary disorders [9, 10]. Pulmonary hypertension in OSA patients can lead to the development of RV hypertrophy and dysfunction. Early studies have reported right heart failure in OSA patients that was associated with daytime hypoxemia due to underlying lung disease [44]. However, recent studies have demonstrated that RV function is impaired in OSA patients even after adjustments for potential confounders [12, 21, 27]. An independent effect of OSA on RV structure and performance was observed in a number of studies, showing a correlation between AHI and RV parameters, such as RV MPI [18, 21, 26], RV EF [12, 21], RV S' [17, 19, 23], RVID [17], RVWT [26], TAPSE [26, 29], and RV strain variables [27, 29, 30]. Another possible mechanism that could lead to RV dysfunction in OSA patients is the generation of negative intrathoracic pressure against an occluded airway, causing increased venous return and volume overload of the right ventricle during apnea periods [29, 45]. During RV hypertrophy, myocardial oxygen demands may increase, leading to RV ischemia and dysfunction [43]. Additionally, OSA is much more common in obese individuals. It has been shown that isolated obesity in young adults is associated with subclinical abnormalities in RV structure and function, which are independent of the presence of OSA and its severity [46, 47].

## 5. Conclusions

The present study represents the first systematic review of the literature and meta-analysis to explore RV involvement in OSA patients. We concluded that OSA patients display increased RV wall thickening and dilatation along with impaired RV function.

## Figures and Tables

**Table 1:** Characteristics of the included studies. RVID: right ventricular basal diameter measured at end-diastole; RVWT: right ventricular wall thickness; RV MPI: right ventricular myocardial performance index; RV S': tricuspid annular systolic velocity; TAPSE: tricuspid annular plane systolic excursion; RV FAC: right ventricular fractional area change; OSA: obstructive sleep apnea; ESS: Epworth Sleepiness Scale; PSG: polysomnography; AHI: apnea-hypopnea index; PAP: pulmonary artery pressure; N/A: not applicable.

Study	Year of publication	Country	Number of participants	Mean (SD) age (years)	BMI (kg/m^2^)	OSA diagnosis (methods; criteria)/control group determination	Mean (SD) ESS score	Mean (SD) AHI	Mean (SD) PAP (mmHg)	RV remodeling and dysfunction measures
Hanly et al. [15]	1992	Canada	31 (OSA)	50 (12.3)	32.8 (7.2)	PSG; AHI < 5 events/h	N/A	49 (25.5)	N/A	RVID, RVWT
			20 (control)	48 (8.8)	26.2 (3.2)	PSG; AHI ≥ 5 events/h	N/A	2.7 (1.6)	N/A	

Guidry et al. [16]	2001	USA	90 (OSA)	60 (9)	32 (5)	PSG; RDI > 90th percentile	N/A	42.0 (15.3) (RDI)	N/A	RV FAC, RVWT, RVID
			90 (control)	61 (9)	28 (4)	PSG; RDI < 50th percentile	N/A	4.6 (2.6) (RDI)	N/A	

Verbraecken et al. [17]	2005	Belgium	43 (OSA)	55 (11)	31.6 (5.4)	PSG; AHI > 20 events/h	N/A	42 (24)	N/A	RVID, RV MPI, RV S'
			40 (control)	50 (16)	26.4 (2.3)	ESS score = 0 (all)	N/A	N/A	N/A	

Dursunoğlu et al. [18]	2005	Turkey	11 (mild OSA)	46.0 (5.6)	30.4 (4.0)	PSG; AHI > 5–< 15 events/h	N/A	25.3 (2.6)	N/A	RVWT, RVID, RV MPI
			18 (moderate-to-severe OSA)	46.5 (4.9)	30.6 (4)	PSG; AHI ≥ 15 events/h	N/A	50.1 (11.6)	N/A	
			20 (control)	43.5 (6)	29.3 (2.4)	PSG; AHI < 5 events/h	N/A	5.2 (2.8)	N/A	

Shivalkar et al. [19]	2006	Belgium	43 (OSA)	55 (11)	31.6 (5.4)	PSG; AHI ≥ 5 events/h	N/A	N/A	32 (10)	RVID, RV MPI, RV S'
			40 (control)	50 (16)	26.4 (2.3)	ESS score = 0 (all)	N/A	N/A	22 (8)	

Kasikcioglu et al. [20]	2007	Turkey	10 (OSA)	42 (6)	30.6 (3.2)	PSG: AHI > 30 events/h	N/A	43.8 (11.7)	N/A	RVID, RV S'
			10 (control)	45 (9)	27.7 (2.6)	Healthy males	N/A	2.1 (1.0)	N/A	

Romero-Corral et al. [21]	2007	USA	15 (mild OSA).	59 (14)	33 (6)	PSG; AHI > 5–< 14 events/h	N/A	9.2 (2.9)	33.6 (12.7)	RV MPI, RV FAC
			36 (moderate-to-severe OSA)	63 (14)	33 (5)	PSG; AHI ≥ 15 events/h	N/A	34.9 (17.9)	33.9 (8.4)	
			26 (control)	55 (15)	31 (6)	PSG; AHI < 5 events/h	N/A	1.7 (1.4)	34.0 (9.4)	

Otto et al. [22]	2007	USA	23 (OSA)	45 (3)	33.7 (0.8)	PSG; AHI ≥ 5 events/h	N/A	50 (7)	N/A	RVID, RV S'
			18 (control)	45 (2)	32.3 (0.9)	PSG; AHI < 5 events/h	N/A	2 (0.4)	N/A	

Tavil et al. [23]	2007	Turkey	20 (OSA)	50 (7)	30 (7)	PSG; AHI ≥ 5 events/h	N/A	31 (29)	N/A	TAPSE, RV S', RV MPI
			21 (control)	49 (5)	29 (6)	PSG; AHI < 5 events/h	N/A	2 (2)	N/A	

Bayram et al. [24]	2008	Turkey	28 (OSA)	44.8 (10.5)	29.7 (5.3)	PSG; AHI ≥ 15 events/h	N/A	62.3 (21.6)	N/A	RVID, RV S',
			18 (control)	41.9 (11.5)	27.9 (2.7)	PSG; AHI < 5 events/h	N/A	2.6 (0.8)	N/A	

Kepez et al. [25]	2009	Turkey	45 (mild-to-moderate OSA)	48.8 (8.2)	28.4 (3.4)	PSG; AHI > 5–< 30 events/h	N/A	15.0 (13.0)	N/A	RVID, RV S'
			40 (severe OSA)	48.6 (9.2)	31.5 (4.9)	PSG; AHI ≥ 30 events/h	N/A	46.0 (42.0)	N/A	
			30 (control)	46.1 (8.7)	27.7 (4.0)	Absence of symptoms of sleep-related disorders	N/A	N/A	N/A	

Tugcu et al. [26]	2009	Turkey	41 (OSA)	56 (12)	31.38 (4.97)	PSG; AHI ≥ 15 events/h, ESS score ≥ 10	19.37 (4.30)	38.84 (21.80)	28.20 (4.66)	RVWT, TAPSE, RV MPI, RV S'
			30 (control)	54 (10)	30.10 (3.65)	PSG; AHI < 5 events/h, ESS score < 10	2.60 (2.29)	1.46 (0.68)	24.73 (2.49)	

Tugcu et al. [27]	2010	Turkey	27 (OSA)	54 (10)	31.1 (5.1)	PSG; AHI ≥ 15 events/h, ESS score ≥ 10	N/A	40 (22)	26.6 (3.1)	RVWT, RVID, TAPSE, RV S'
			26 (control)	54 (10)	29.6 (3.6)	PSG; AHI < 5 events/h, ESS score < 10	N/A	2 (1)	24.1 (2.3)	

Yang et al. [28]	2012	China	77 (mild OSA)	58.4 (0.7)	27.39 (5.74)	PSG; AHI 5–15 events/h, SpO_2_ nadir 85–89%	N/A	20 (5.6)	28 (6)	RVID, RVWT, RV MPI,
			81 (moderate OSA)			PSG; AHI 15–30 events/h, SpO_2_ nadir 80–84%	N/A		38 (6)	
			62 (severe OSA)			PSG; AHI > 30 events/h, SpO_2_ nadir <80%	N/A		55 (11)	
			75 (control)	59.8 (1.1)	26.32 (4.57)	PSG; AHI < 5 events/h	N/A	2.9 (2)	22 (5)	

Altekin et al. [29]	2012	Turkey	20 (mild OSA)	46.95 (6.4)	28.68 (3.44)	PSG; AHI > 5–< 15 events/h	N/A	10.73 (2.57)	27.62 (4.32)	RV MPI, TAPSE
			19 (moderate OSA)	46.79 (5)	29.05 (2.26)	PSG; AHI > 15–< 30 events/h	N/A	20.52 (2.60)	29.78 (4.24)	
			19 (severe OSA)	46.68 (7.6)	29.80 (2.38)	PSG; AHI ≥ 30 events/h	N/A	58.1 (16.27)	32.06 (3.71)	
			21 (control)	45.38 (4.5)	26.35 (4.14)	ESS score < 10	N/A	N/A	25.61 (5.43)	

Hammerstingl et al. [30]	2012	Germany	154 (OSA)	61.7 (12.4)	31.1 (5.8)	PSG; AHI < 5 events/h	10.4 (5.2)	35.9 (28.4)	22.0 (12.6)	TAPSE, RV MPI
			29 (control)	55.7 (15.8)	30.1 (5.5)	PSG; AHI ≥ 5 events/h	7.9 (5.6)	2.3 (1.3)	17.3 (11.9)	

Öztuna et al. [31]	2013	Turkey	23 (OSA)	53.6 (7.1)	34.8 (7.8)	PSG; AHI ≥ 15 events/h	9.17 (5.10)	N/A	21.8 (5.9)	RV MPI
			23 (control)	53.7 (6.5)	25.8 (1.7)	ESS score < 10	0.26 (0.54)	N/A	22.1 (4.4)	

Araz et al. [32]	2014	Turkey	67 (OSA)	49.7 (12.7)	34.6 (8.3)	PSG; AHI ≥ 5 events/h	10 (4)	52 (6)	41.2 (12.9)	RVID
			31 (control)	49.8 (10.9)	29.5 (7.3)	PSG; AHI < 5 events/h	3 (2)	1.5 (1.4)	25.3 (7.5)	

Vitarelli et al. [33]	2015	Italy	10 (mild OSA)	47.9 (10.3)	26.9 (5.8)	PSG; AHI > 5–< 15 events/h	N/A	7.1 (1.9)	25 (6)	TAPSE, RV MPI, RV FAC, RV S'
			8 (moderate OSA)	47.6 (9.1)	27.4 (5.5)	PSG; AHI > 15–< 30 events/h	N/A	19.8 (2.7)	35 (11)	
			19 (severe OSA)	48.1 (10.2)	28.2 (6.3)	PSG; AHI ≥ 30 events/h	N/A	58.9 (9.1)	46 (11)	
			30 (control)	46.2 (13.4)	26.4 (4.3)	PSG; AHI < 5 events/h	N/A	3.8 (1.4)	22 (3)	

Ozkececi et al. [34]	2016	Turkey	30 (OSA)	49.6 (11.7)	31.6 (5.8)	PSG;AHI ≥ 5 events/h	N/A	24.5 (6–98)	22.4 (8.9)	RVID, RV MPI, RV S', TAPSE
			60 (control)	46.4 (14)	29.3 (4.8)	PSG; AHI < 5 events/h	N/A	1 (1–4)	12.7 (7.5)	

Altiparmak et al. [35]	2016	Turkey	52 (OSA)	49 (10)	26.5 (2.1)	PSG; AHI ≥ 5 events/h	N/A	N/A	32.4 (7)	RVID, RVWT, TAPSE, RV S'
			42 (control)	46(7)	25.8(3.3)	PSG; AHI < 5 events/h	N/A	N/A	21.6 (4.3)	

Li et al. [36]	2016	China	24 (mild OSA)	47.3 (6.1)	26.40 (3.12)	PSG; AHI > 5–< 15 events/h	N/A	12.72 (2.03)	N/A	RVWT, RVID, TAPSE, RV FAC
			25 (moderate OSA)	47.9 (7.9)	26.83 (3.55)	PSG; AHI > 15–< 30 events/h	N/A	24.01 (3.56)	N/A	
			20 (severe OSA)	48.5 (5.4)	27.97 (3.59)	PSG; AHI ≥ 30 events/h	N/A	40.78 (5.02)	N/A	
			31 (control)	46.8 (5.4)	24.86 (2.78)	PSG; AHI < 5 events/h	N/A	1.72 (1.01)	N/A	

Dobrowolski et al. [37]	2016	Poland	45 (mild OSA)	47.5 (10.5) All	N/A	PSG; AHI > 5–< 15 events/h	N/A	N/A	N/A	TAPSE, RV FAC, RV S'
			27 (moderate OSA)		N/A	PSG; AHI > 15–< 30 events/h	N/A	N/A	N/A	
			40 (severe OSA)		N/A	PSG; AHI ≥ 30 events/h	N/A	N/A	N/A	
			43 (control)		N/A	PSG; AHI < 5 events/h	N/A	N/A	N/A	

Zhou et al. [38]	2016	China	21 (mild OSA)	52.8 (11.9)	29.6 (9.1)	PSG; AHI > 5–< 15 events/h	N/A	N/A	35.7 (6.1)	RV FAC, RV MPI
			19 (moderate OSA)	55.7 (12.4)	35.2 (10.1)	PSG; AHI > 15–< 30 events/h	N/A	N/A	33.4 (5.8)	
			23 (severe OSA)	50.2 (12.1)	39.2 (9.8)	PSG; AHI ≥ 30 events/h	N/A	N/A	18.7 (6.9)	
			19 (control)	52.0 (10.8)	24.2 (3.7)	PSG; AHI < 5 events/h	N/A	N/A	16.7 (6.2)	

D'Andrea et al. [39]	2016	Italy	55 (OSA)	67.8 (11.2)	33.6 (6.6)	PSG; AHI ≥ 5 events/h	N/A	35.1 (15.4)	35.8 (14.4)	RVID, RVWT, TAPSE
			45 (control)	65.9 (12.3)	28.7 (3.6)	Absence of cardiovascular, structural, and functional abnormalities	N/A	N/A	24.8 (11.3)	

**Table 2:** Results of the meta-analysis comparing OSA patients and healthy controls. RVID: right ventricular basal diameter measured at end-diastole; RVWT: right ventricular wall thickness; RV MPI: right ventricular myocardial performance index; RV S': tricuspid annular systolic velocity; TAPSE: tricuspid annular plane systolic excursion; RV FAC: right ventricular fractional area change; OSA: obstructive sleep apnea; WMD: weighted mean difference; CI: confidence interval.

Echocardiographic parameters	Number of studies	OSA/control	WMD (95% CI)	*p* value	Study heterogeneity			Egger's test *p* value
					*I* ^2^	*χ* ^2^	*p* value	
RVID	16	902/596	2.49 (1.62, 3.37)	0.000	96.8%	647.61	0.000	0.001
RVWT	9	579/397	0.82 (0.51, 1.13)	0.000	95.6%	344.56	0.000	0.671
RV MPI	14	864/434	0.08 (0.06, 0.10)	0.000	84.1%	157.39	0.000	0.150
RV S'	14	639/391	−0.95 (−1.59, −0.32)	0.003	88.4%	190.21	0.000	0.347
TAPSE	11	655/378	−1.76 (−2.73, −0.78)	0.000	89.3%	187.24	0.000	0.462
RV FAC	6	422/239	−3.16 (−5.60, −0.73)	0.011	80.2%	70.83	0.000	0.006
